# Malignancy diagnosis of liver lesion in contrast enhanced ultrasound using an end-to-end method based on deep learning

**DOI:** 10.1186/s12880-024-01247-y

**Published:** 2024-03-21

**Authors:** Hongyu Zhou, Jianmin Ding, Yan Zhou, Yandong Wang, Lei Zhao, Cho-Chiang Shih, Jingping Xu, Jianan Wang, Ling Tong, Zhouye Chen, Qizhong Lin, Xiang Jing

**Affiliations:** 1 The Third Central Hospital of Tianjin, 83 Jintang Road, Hedong District, Tianjin, 300170 China; 2 Tianjin Key Laboratory of Extracorporeal Life Support for Critical Diseases, Tianjin, China; 3 Artificial Cell Engineering Technology Research Center, Tianjin, China; 4 Tianjin Institute of Hepatobiliary Disease, Tianjin, China; 5 Philips Ultrasound R&D Research, Shanghai, China

**Keywords:** Contrast enhanced ultrasound (CEUS), Liver lesion, Malignancy diagnosis, Deep Learning

## Abstract

**Background:**

Contrast-enhanced ultrasound (CEUS) is considered as an efficient tool for focal liver lesion characterization, given it allows real-time scanning and provides dynamic tissue perfusion information. An accurate diagnosis of liver lesions with CEUS requires a precise interpretation of CEUS images. However,it is a highly experience dependent task which requires amount of training and practice. To help improve the constrains, this study aims to develop an end-to-end method based on deep learning to make malignancy diagnosis of liver lesions using CEUS.

**Methods:**

A total of 420 focal liver lesions with 136 benign cases and 284 malignant cases were included. A deep learning model based on a two-dimensional convolution neural network, a long short-term memory (LSTM), and a linear classifier (with sigmoid) was developed to analyze the CEUS loops from different contrast imaging phases. For comparison, a 3D-CNN based method and a machine-learning (ML)-based time-intensity curve (TIC) method were also implemented for performance evaluation.

**Results:**

Results of the 4-fold validation demonstrate that the mean AUC is 0.91, 0.88, and 0.78 for the proposed method, the 3D-CNN based method, and the ML-based TIC method, respectively.

**Conclusions:**

The proposed CNN-LSTM method is promising in making malignancy diagnosis of liver lesions in CEUS without any additional manual features selection.

## Introduction

Hepatocellular carcinoma (HCC) is the most common primary liver malignancy and the 3rd most common cause of cancer-related death worldwide, which occupied 70–90% of various kinds of primary liver cancers [1]. Unfortunately, most patients with HCC are diagnosed at the advanced stage and unsuitable for surgery or local treatment, which leads to poor prognosis with median overall survival (OS) of about 6 months [2]. Therefore, an accurate diagnosis for distinguishing HCC from focal liver lesions (FLLs) at the early stage is essential.

Contrast-enhanced ultrasound (CEUS) is considered as an effective tool in the characterization of focal liver lesions [3]. Compared to Contrast-enhanced computed tomography (CE-CT) and Contrast-enhanced Magnetic Resonance Imaging (CE-MRI), CEUS operates in real-time with higher temporal resolution and can provide dynamic information on tissue perfusion during all phases of enhancement. During the CEUS examination, clinicians usually need to carry out a series of tasks with tedious details including size measurements, enhancement characteristics analysis while monitoring the tissue perfusion [4]. An accurate and reliable diagnosis of liver lesions with CEUS is based on a well-defined and precise interpretation of the CEUS loops. However, this is a highly experience dependent task and it requires significant amount of training and practice for a clinician to become proficient. Indeed, clinicians skilled in CEUS diagnosis are limited, in particular in lower level hospitals. To some extent, this issue confines the widespread of CEUS application in Liver. Nonetheless, recent advances in computer aided diagnosis may has the potential to reduce the operator-dependency and to popularize the application of CEUS.

Generally, CAD methods to facilitate CEUS mode based diagnosis can be divided into two categories, the classic machine learning methods relying on feature engineering and the deep learning methods that are capable of feature learning. In tradition machine learning methods, some works were based on perfusion features extracted from the time intensity curve (TIC) [5–7].For example, in [5], the authors used sparse non-negative matrix factorization to automatically extract the TIC based quantitative parameters and employed a neural network classifier to classify benign and malignant liver lesions with an accuracy of 86.36%. Similar efforts have been made in [6], where authors extracted both images features from B-mode and CEUS images and perfusion features from TICs then fed into Support Vector Machine (SVM) and Artificial Neural Networks (ANN) classifiers to make the class prediction. The reported accuracy of the SVM method was 81.1% in benign/malignancy classification and was similar that of an expert reader, 81.4%. In [7], 28 features were extracted from TICs in the CEUS cine-loops from a case were used as an input to SVM for benign/malignancy classification with an accuracy of 91.8%. Nevertheless, these TIC based methods depend on manual ROI selections for TIC generation, which can be subjective to motion impacts.Other classic machine learning methods could include extract CEUS image features as input. In [8], the authors proposed to manually select three frames from CEUS cine loops during arterial phase (AP), portal venous phase (PVP), and late phases (LP), respectively. Using these images, they extracted tens features and applied a multiple kernel learning classifier to predict liver lesion malignancy (Guo et al,  [8]). Indeed, either the selection of CEUS frames, ROIs, or hand-crafted features can be highly operator experienced dependent, time consuming, and thus limiting the classification performance.

On the other hand, deep learning (DL) methods outperformed many traditional feature engineering based machine learning methods. The advantages of deep learning (DL) are mainly lies in threefold. First, deep learning can automatically uncover features from the training data, hence significantly alleviate efforts of hand-crafted features. The learned features may compensate and even surpass the discriminative power of the conventional feature extraction methods. Second, with deep learning, feature interaction and hierarchy can be exploited jointly within the intrinsic deep architecture of a neural network. Consequently, the feature selection process will be significantly simplified. Third, three steps of feature extraction, selection, and supervised classification can be realized within the optimization of the same deep architecture. This systematic design fashion makes the model easier to be fine-tuned for achieving better performance.

Various DL algorithms have been proposed to facilitate the assessment of focal liver lesions (FLLs) dignity and/or entity classifications in ultrasound using B-mode and/or CEUS image data [9, 10]. DL algorithms developed based on B-mode data typically only use static images as data sources. As such, only spatial information is taking into account and the developednetworks are primarily VGG-type 2D CNN, DenseNet 2D CNN, and ResNet-type 2D CNN [11–15]. Unlike B-mode, CEUS provides dynamic perfusion information on the tissue in real-time. CEUS images and cine-loops during the examination contain both spatial and temporal information. Intuitively, DL methods applies to CEUS data for liver lesion classification should have the potential to simultaneously obtain spatio-temporal information. In [16], authors proposed to use a general deep learning-based method to predict personalized responses of HCC to first transarterial chemoembolization (TACE) sessions. They utilized a 3D-CNN model to extract spatio-temporal information from CEUS data and showed it performed better than a pre-defined TIC feature-based model and a pre-defined radiomics feature-based model. Although the objective of this study [16] was not to predict the malignancy of liver lesions, it still demonstrated the robust performance and the potential of adopting deep learning to CEUS for liver applications. In [17], the authors proposed a 3D-CNN based DL network classifiy benign and HCC tumors. In their work, a fixed number of CEUS video frames was selected with a uniform time sampling and then used to form a 3-dimensional data. Finally, the spatial and temporal features were extracted simultaneously using the 3D-CNN. However, fusing CEUS temporal dimension and spatial information resulting losing the time-varying information during contrast perfusion, which is critical for lesion classification. In [18], the authors proposed a model based on transfer learning to obtain image features from CEUS images at different perfusion phases for liver lesion classification. In [19] CEUS frames were manually selected from CEUS videos at different perfusion phases then fed to multiple parallel 2D ResNet networks to extract features and fuse them in a fully connected layer, which was used to classify benign and malignant images. However, this approach didn’t consider the correlation between perfusion phases. This is again very important for physicians to make diagnosis. A similar parallel multi-network framework was also proposed in [20], this differences between the two works lies in different of number of CEUS video frames selection and different ResNet network chosen. In [21], the authors proposed a framework containing a ResNet network followed by a Views-Related Learning module (VRL) and a Two-Step-Orthogonal-Projection (TSOP) module. The ResNet network served as backbone to extract features from manually selected CEUS video frames from various perfusion phases and the VRL+TSOP was used to learn and fuse dynamic temporal features. In [22], the authors proposed to use a 2D-CNN + LSTM model to classify HCC from FNH based on manually selected CEUS video frames. The LTSM model provides the feasibility to learn temporal correlation between perfusion phases in addition to spatial information extraction using 2D-CNN. However, the current work, only considered CEUS videos from arterial phase. Information from the portal venous and late phases was missing but was critical during liver lesion benign and malignancy classification. Nonetheless, all algorithms above requires experienced radiologist to manually select CEUS video frames from different perfusion phases, which again would be operator-dependent. In this manuscript, we propose an end-to-end DL method using CNN-LSTM model to distinguish between benign and malignant liver lesions using CEUS video cine-loops from all perfusion phases, i.e. AP, PVP and LP We aim to avoid as many manual interactions needed from radiologist as possible to make classification more objective and automated. To our knowledge, our work is the first attempt of applying CNN-LSTM model in CEUS liver lesion diagnosis without any additional manual features selection.

This paper is organized as follows. In Materials and methods section, we presented the data and described the methods in detail. Results of the proposed method in comparison with a classical 3D-CNN model and a ML TIC-based method are provided in Results section. The conclusion are drawn in Discussion section.

## Materials and methods

### Dataset

**Fig. 1:**
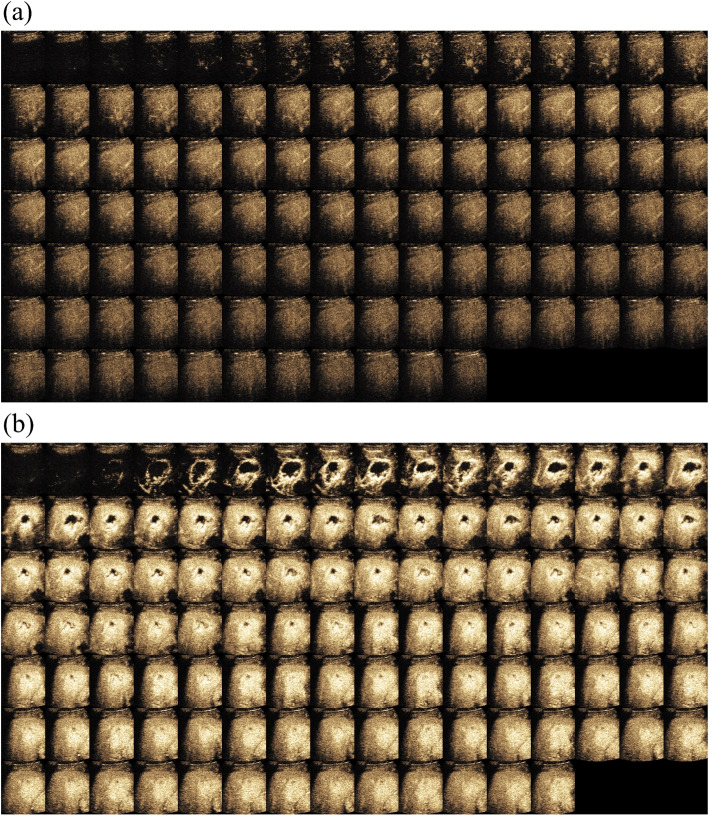
CEUS sequences of two typical cases. Each small-size CEUS image was selected from the CEUS loop at one second interval starting from AP onset time. **a **CEUS frames of a typical malignant liver lesion. **b** CEUS frames of a typical benign liver lesion

This is a retrospective study and was approved by the Institutional Review Board and Ethics Committee. The requirement for informed consent was waived off. From May 2018 to May 2019, 440 patients with focal liver lesion who underwent CEUS examination were recruited. The CEUS examinations were taken using different ultrasound imaging systems (Philips, Siemens, Mindray and Canon medical systems). During the examination, a bolus of 1.5-2.4 ml of Sonovue (Bracco, Milan, Italy) was injected intravenously through a cubital vein, followed by a flush of NaCl 0.9% 5 ml in bolus. Real-time side by side contrast-enhanced mode was turned on with the mechanical index (MI) was set as low as possible at range of 0.05 to 0.1. Usually, clinicians record several loops from the end of contrast agent injection to about 6 minutes. All CEUS loops were stored in DICOM format. Each frame in loop had both CEUS data and B-mode data in dual display with resolution in 960 (Height) x 1280 (Width) x 3 (RGB). Given this was an retrospective study, the imaging parameter settings on different ultrasound systems were not necessarily the same. Ultrasound doctors adjusted the parameters while scanning to obtain the optimal image quality for them to make the diagnosis. This is more close to common situation during typical clinical practice. In Fig. 1, we showed two typical cases in CEUS.

**Fig. 2:**
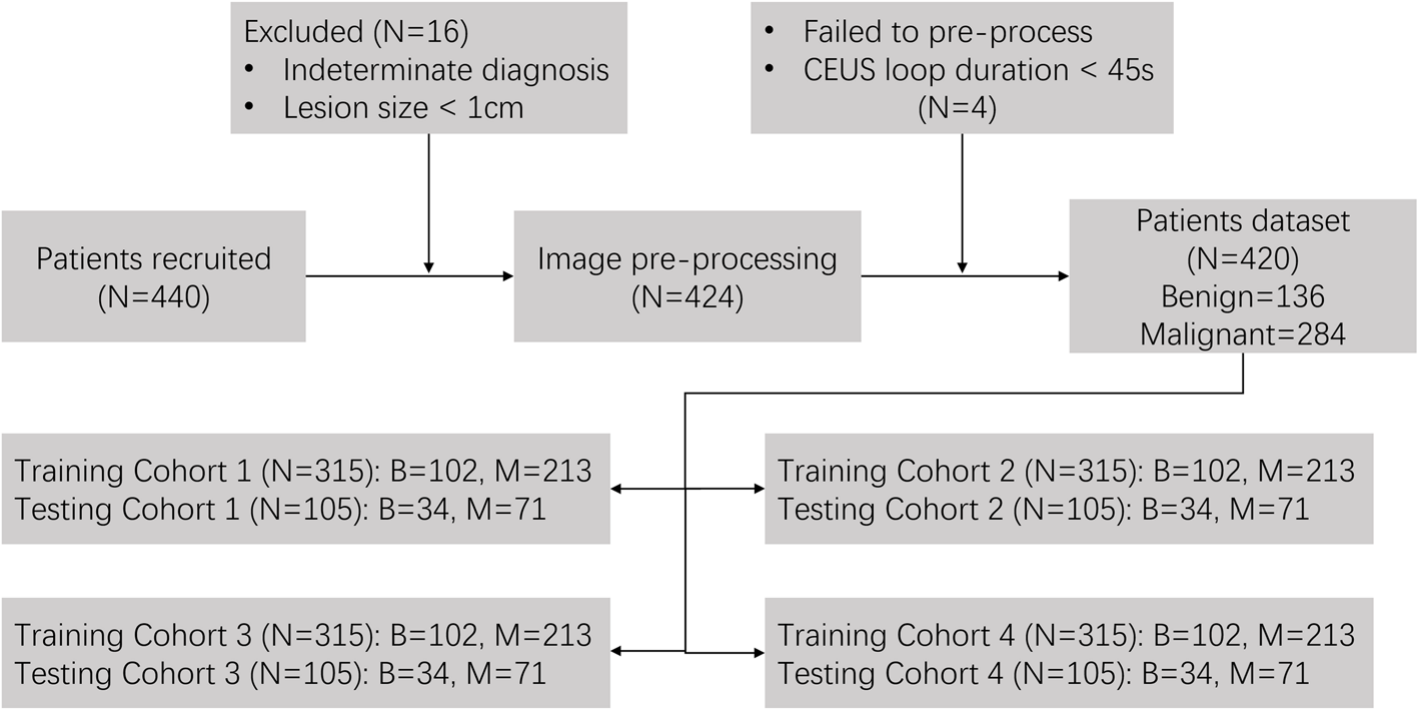
The inclusion and exclusion criteria for patient cohort

From the 440 patients above, we further selected with inclusion and exclusion criteria as below. Inclusion criteria were as follows: 1) aged 18-80 years old; 2) diagnosis confirmed by histologic examination, EASL guidelines, contrast MRI scanning, or follow-up beyond 6 months; 3) CEUS loops were completed in the sense that all three phases including AP, PVP and LP were recorded. Exclusion criteria were as follows: 1) lesion size $\le$ 1cm; 2) lack of dicom header or fail to load and preprocess; 3) Duration of the first CEUS loop is shorter than 45s. Finally, 420 patients including 136 benign and 284 malignant cases were enrolled in our study, shown as Fig. 2. Table 1 indicates the amounts of benign and malignant cases with their corresponding subtypes. Patients were randomly divided into training and testing cohorts with 3:1 ratio (4-fold cross-validation) for algorithm development and evaluation.

**Table 1:** Amount of the benign and malignant cases with their corresponding subtypes

Type	Subtype	Amount	Diagnoses Methods
Benign	HA	55	Histologic examination: 20 cases
			Clinic follow-up: 35 cases
	HEM	60	Histologic examination: 35 cases
			Contrast MRI scanning: 25 cases
	FNH	13	Histologic examination
	RN	8	Histologic examination
Malignant	HCC	257	Histologic examination: 94 cases
			^a^EASL guidelines [23] : 163 cases
	IHCC	7	Histologic examination
	HM	20	Histologic examination

*Abbreviations*: *HA *Hepatic abscess, *HEM *Hemangioma, *FNH *Focal nodular hyperplasia, *RN *Regenerative nodule, *HCC *Hepatocellular Carcinoma, *IHCC* Intrahepatic Cholangiocarcinoma, *HM *Hepatic Metastasis

^a^The diagnosis was confirmed by following the EASL guidelines

### Method

In this section, we will introduce the proposed method and two methods for comparison.

#### Proposed method

**Fig. 3:**
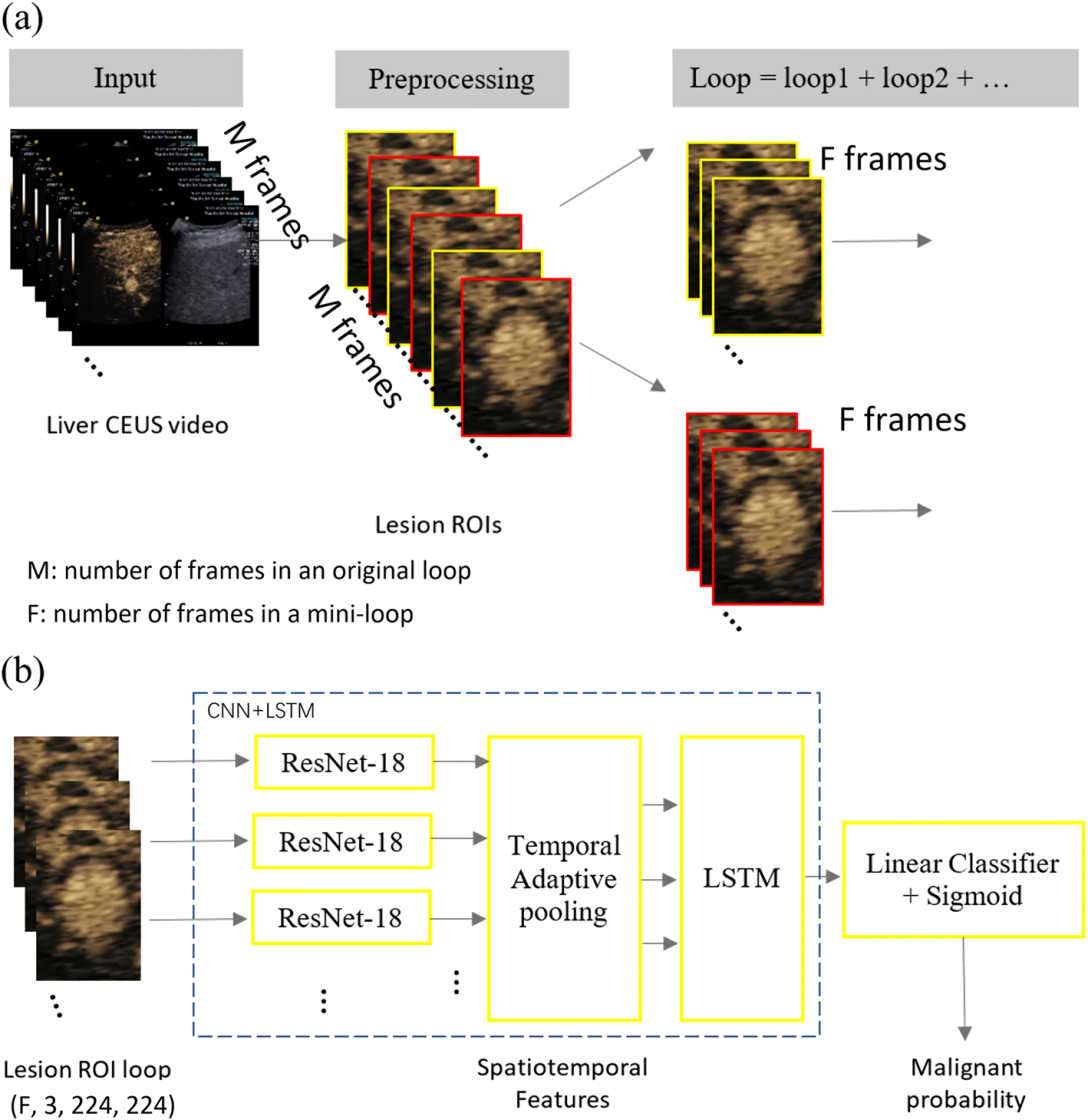
Flowchart of the proposed method. **a** Flowchart of the pre-processing step. **b** Flowchart of the CNN-LSTM mode

The most intuitive way to employ deep learning technique in the diagnosis of liver lesion with CEUS is to feed the CEUS loops into a 3D CNN model because 3D CNN preserves both spatial and temporal features. However, the 3D CNN has high computational complexity. With limited number of datasets, the model can be easily overfitted. To overcome this issue, we proposed to use the CNN-LSTM model to automatically make the differential diagnosis of liver lesion based on CEUS loops. In this method, CNN extracts spatial features of each frame, followed by the LSTM to learn the temporal features from the sequences of these spatial features. The flowchart of the method is shown in Fig. 3 and the detailed description are as follows.

##### Crop Region of Interest Sequence (ROIs) of liver lesion.

Generally in clinical practice, the clinician would label four markers to denote long and short axis of lesion in a selected frame, which can be utilized as the bounding box of lesion. To reduce the effect of respiratory motion, the size of this bounding box is expanded by 50%.

##### Split whole CEUS cine-loops into several mini-loops.

As mentioned, an expert clinician can give reliable and accurate diagnosis results for liver lesions mainly based on typical CEUS frames from AP, PVP and LP. Leveraging this domain knowledge, we selected F (e.g. F=4) frames at equal time intervals from each phase (AP, PVP, LP) to reduce the impact from out of plane frames which were caused by respiratory motion. The removal work was automatic with no visual inspection or manual interaction required from a radiologist. It was done through a TIC curve fitting based method. For a given frame, if the absolute difference between its average intensity and the TIC fitted intensity was larger than 2 times of the standard deviation of the average intensity of all frames, it was removed. Since the duration of three phases are not the same, different number of F-frame may be generated. As suggested by guidelines such as CEUS LI-RADS [23], the last few seconds of PVP and LP are the most important frames. With this prior information, we select F frames from back to front on the timeline of PVP and LP. Finally, the entire CEUS loop from each patient was split into multiple mini-loops and each of which has a malignancy probability as the output of the proposed network. The final prediction result is generated by the majority vote of mini-loops. Compared with using the whole loop directly, splitting each CEUS loop into multiple mini-loops can increase sample size and mitigate the risk of overfitting. This can increase the total number of CEUS loops inputting into the network, which in principle equivalent to augument the data temporally.

##### Map each mini-loop to malignant probability with CNN-LSTM model.

We employed residual networks because of its good performance and simplicity. Each ROI image feeds to the ResNet-18 [24] network (ResNet) to extract spatial features. The ResNet18 was pretrained based on ImageNet by freezing the first 5 layers. Then the F*3 dimensional spatial features of mini-loop are smoothed to be 3 dimensional via temporal adaptive pooling in order to mimic the features from AP, PVP and LP and to reduce the respiratory motion at the same time. Afterwards, 3 dimensional spatial features are fed to the LSTM [25] to extract spatio-temporal features through adaptive average pooling layer. The LSTM contains two layers. The first layer is bidirectional LTSM to have sequence information in both directions backwards and forwards. The number of features in the hidden state is 128. Finally spatio-temporal features from every mini-loop are used to predict malignancy probability.

#### Methods for comparison

In this paper, we compared the proposed method with a 3D-CNN based method and a classical ML-based TIC method.

##### 3D-CNN based method.

As mentioned, the most intuitive way to process video based data is to employ a 3D-CNN. With limited number of datasets as well as memory, one can not directly input the CEUS loop into a 3D-CNN. Thus, the same pre-processing step as Fig. 3a was used. Instead of using CNN+LSTM as Fig. 3b, 3D-CNN was employed in this method.

##### ML-based TIC method.

**Fig. 4:**
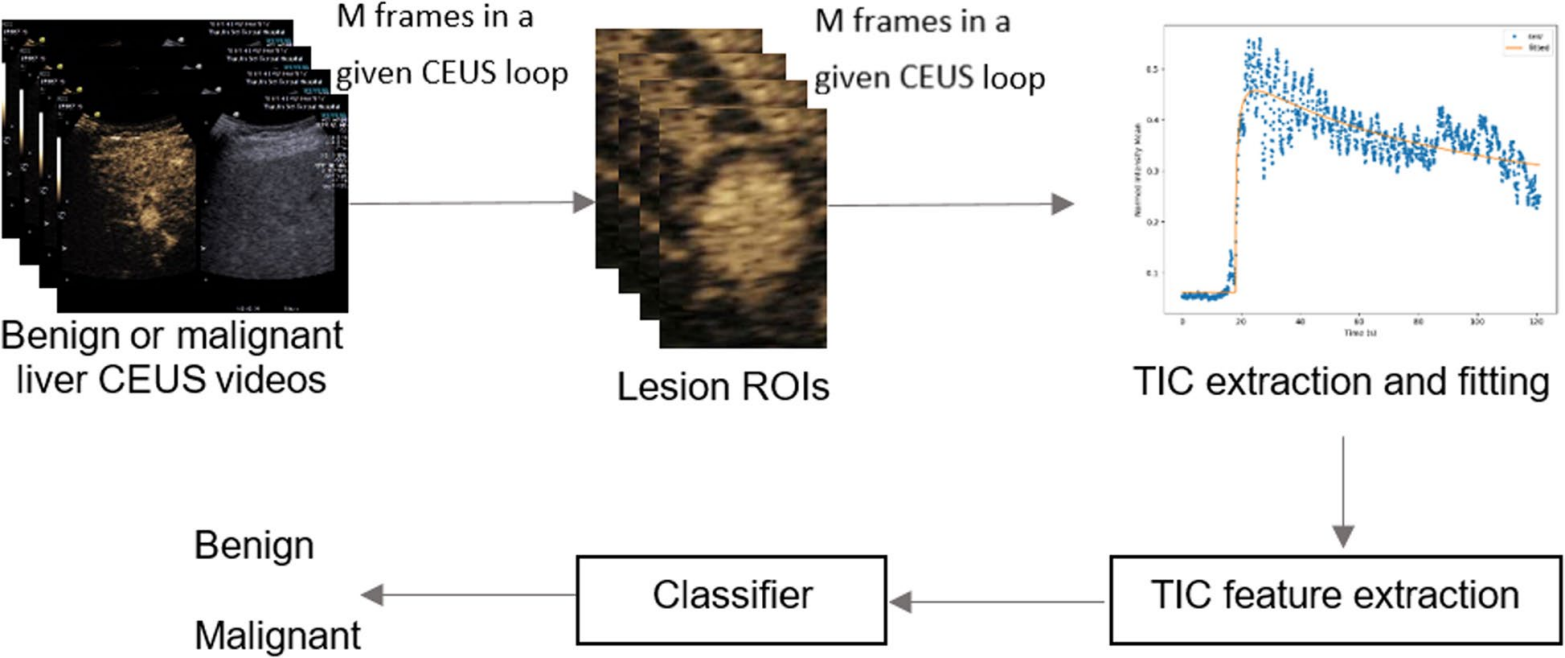
Diagram of ML-based TIC method

The block diagram of this method is shown in Fig. 4. TIC was firstly generated using the signal intensity mean value of a manually drawn ROI within the lesion. Due to respiratory motion, the TIC is usually very noisy and curve fitting is required. There are five different perfusion models commonly used: the lognormal distribution, the gamma variate function, local density random walk (LDRW), first passage time (FPT), and the lagged normal function [26]. In practice, there is no standards or guideline for choosing the best perfusion model. Moreover, using single perfusion model to estimate perfusion parameters is very sensitive to either the initial values for perfusion parameters or the selection of appropriate boundary conditions. Therefore, we have implemented a Multi-Model Framework, which applies nonlinear regression optimizing method to all five perfusion models. Afterwards, the perfusion parameters were chosen from the model with the lowest estimation error by evaluating various curve-fitting models and selecting the optimal solution using an exhaustive search technique. This framework was implemented in-house in Python. The perfusion parameters include AP onset time, peak time, peak intensity, area under the curve, mean transit time, gradient of wash in, gradient of wash out. Finally, all of these parameters plus the lesion size were input into a classifier. XGBoost was used as the classifier and it was chosen based on experiments with best results.

In this work, a NVIDA GPU GeForce GTX 1080 Ti 11 GB was adopted to train different models. K-fold cross-validation (for example: k=4 here) method was performed to avoid the sampling bias by randomly partitioning dataset stratified by benign and malignant. For the proposed and 3D-CNN based method, models were trained by a stochastic gradient descent solver with a cross-entropy loss function for 6 epochs in total, with learning rate 0.01 and 0.001 in the first 4 epochs and in the last 2 epochs.

#### Evaluation Metrics

In this study, four evaluation metrics were used to evaluate the model performance, namely: receiver operating characteristic (ROC) curve, the area under the curve (AUC), sensitivity and specificity. Sensitivity and specificity can be calculated as follows:

1 $$\begin{aligned} sensitivity = \frac{TP}{TP+FN} \end{aligned}$$

2 $$\begin{aligned} specificity = \frac{TN}{TN+FP} \end{aligned}$$

TP, TN, FP and FN represent the number of true positives, true negatives, false positives and false negatives.

## Results

**Fig. 5:**
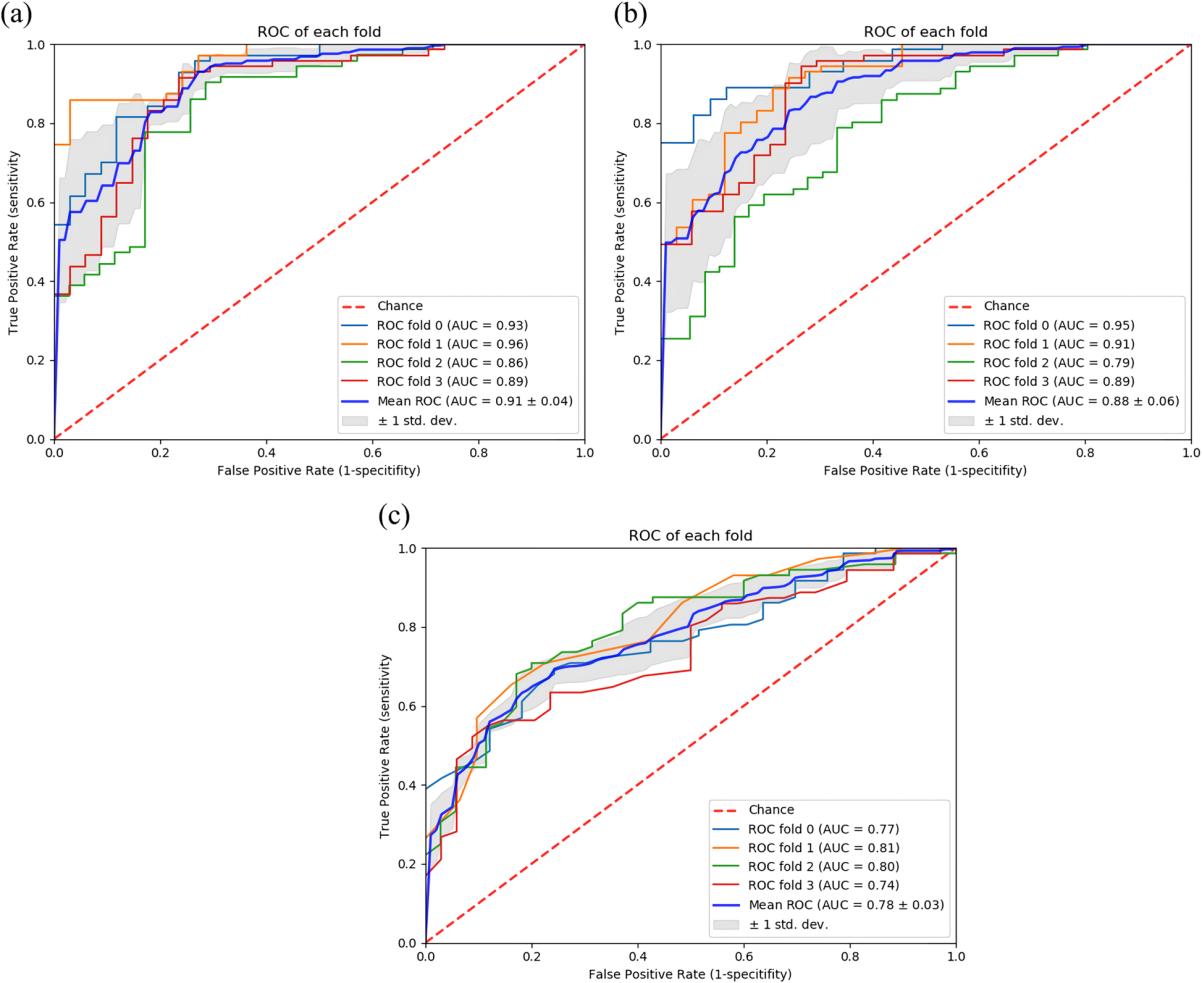
ROC curves of the proposed method, 3D-CNN based method and ML-based TIC method with fold parameter ranging from 0 to 3. **a** Proposed method. **b** 3D-CNN based method. **c** ML-based TIC method

The area under the receiver operation characteristic (ROC) curve (AUC) with 4-fold cross-validation was adopted as the quantitative metrics for evaluating the performance of the proposed algorithm for distinguishing between benign and malignant liver lesions. Mean AUCs and their corresponding standard deviations for each fold validation are shown in Fig. 5. The results demonstrate that the mean AUC evaluated is 0.91, 0.88, and 0.78 for the proposed CNN-LSTM based method, the 3D-CNN based method and the ML-based TIC method, respectively. According to previous studies in literature, the AUC for an experienced observer (more than 20 years of performing and interpreting CEUS) and an inexperienced observer (clinician without prior CEUS experience) to make malignancy diagnosis of liver lesions was 84% and 72%, respectively [6]. Although the datasets are different, this result once again confirmed that the deep learning based methods are promising in providing computer aided diagnosis for liver lesion in CEUS. More specifically, we show in Table 2 the changes in sensitivity and specificity of the three methods when applying different thresholds for the predicted malignancy probabilities. The sensitivity results increased from 0.83 to 0.95 and the specificity decreased from 0.82 to 0.7 with the threshold by using the purposed CNN-LSTM model. The 3D-CNN based method shows the highest sensitivity of 0.96, but with the specificity of 0.55; the ML-based TIC method also shows the lowest specificity of 0.21 when the sensitivity is 0.96.

**Table 2:** The sensitivity and specificity of three methods. Operator 1, 2, 3 represent different thresholds of the predicated malignant probability

Method/(Sensitivity, Specificity)	Operator 1	Operator 2	Operator 3
CNN-LSTM	(0.83, 0.82)	(0.91, 0.74)	(0.95, 0.70)
3D CNN	(0.83, 0.74)	(0.91, 0.67)	(0.96, 0.55)
TIC based	(0.87, 0.42)	(0.90, 0.36)	(0.96, 0.21)

## Discussion

This paper demonstrated an end-to-end methodology from data preprocessing to model implementation to successfully make computer aided diagnosis of liver lesions in CEUS. Compared with the traditional ML-based TIC method, the most intuitive 3D CNN based method used as benchmark in this work and the previous literatures [9, 10], it does not require any additional manual features selection and CEUS frames selection but has a higher AUC. With the proposed method, the only manual input that a clinician may need to do is to draw a ROI to indicate the lesion when he/she doesn’t measure the lesion before entering CEUS mode, then the lesion benign/malignancy will be automatically prediction by the proposed method. The main contributions of this proposed method include 1) Automatically sample frames from the entire CEUS loops leveraging clinical domain knowledge, cascade them into mini-loops as the input of the model, providing dynamic information of CEUS in a more effective way. 2) An efficient variant model of CNN, i.e. CNN-LSTM, was applied to avoid the potential overfitting problem and to extract spatio-temporal features. As far as we know, this is the first time to employ CNN-LSTM in analysis whole contrast enhanced ultrasound video cine-loops (i.e., including all CEUS perfusion phases, AP, PVP and LP). 3) A temporal adaptive pooling is designed and added in the model to reduce the impact of respiratory motion significantly.

Furthermore, we invited three clinicians with 5, 8 and 10 years of CEUS experience to analyze the misdiagnosed cases predicted from the proposed method. Eight misdiagnosed cases were selected and got in-depth reviews, including four benign cases (misdiagnosed as malignancy) and four malignant cases (misdiagnosed as benign). Among the eight cases, two FNH cases were presented with mild washout in the LP, two HA cases showed fast wash in and fast wash out and two HCC cases demonstrated with classical hyper enhancement in AP but lack of markable washout during LP. All three clinicians confirmed that these patterns are actually atypical in clinical. In other words, six of them presented with features that does not usually belong to their own class. The remaining two cases are HM, which has the least number of cases in the training data. To conclude, it is noted that all of above cases were considered difficult to diagnosis even for an experienced clinician when only CEUS loops are provided.

We should mention that clinicians usually make a diagnosis decision based on comprehensive information including patient’s medical history, related surgical examination, and even medical images from other modalities as well. We also noticed that there are some promising results using regular B-mode [11, 27]. Therefore, our future work include: 1) Increase the variability of dataset including more cases such as HM; 2) Combine patient’s information not only in CEUS; 3) compare with the performance of clinicians with different level of experience.

## Conclusions

The study successfully developed an end-to-end deep learning algorithm to classify the liver lesions between benign or malignant effectively. The proposed algorithm with promising results has a high potential to narrow the gap in liver lesion diagnosis between radiologists with different experience levels. Once the proposed model with convincing results is translated into clinical workflow, we believe that it can become a second opinion for the radiologist or the ultrasound doctor to help them make a precise diagnosis.

## Data Availability

The datasets in this study are not publicly available due to institutional review board.
